# Anisotropy of Photopolymer Parts Made by Digital Light Processing

**DOI:** 10.3390/ma10010064

**Published:** 2017-01-13

**Authors:** Mario Monzón, Zaida Ortega, Alba Hernández, Rubén Paz, Fernando Ortega

**Affiliations:** 1 Departamento de Ingeniería Mecánica, Universidad de Las Palmas de Gran Canaria, Las Palmas de Gran Canaria 35017, Spain; alba.hernandez104@alu.ulpgc.es (A.H.); ruben.paz@ulpgc.es (R.P.); fernando.ortega@ulpgc.es (F.O.); 2 Departamento de Ingeniería de Procesos, Universidad de Las Palmas de Gran Canaria, Las Palmas de Gran Canaria 35017, Spain; zaida.ortega@ulpgc.es

**Keywords:** additive manufacturing, photopolymer, digital light processing, anisotropy

## Abstract

Digital light processing (DLP) is an accurate additive manufacturing (AM) technology suitable for producing micro-parts by photopolymerization. As most AM technologies, anisotropy of parts made by DLP is a key issue to deal with, taking into account that several operational factors modify this characteristic. Design for this technology and photopolymers becomes a challenge because the manufacturing process and post-processing strongly influence the mechanical properties of the part. This paper shows experimental work to demonstrate the particular behavior of parts made using DLP. Being different to any other AM technology, rules for design need to be adapted. Influence of build direction and post-curing process on final mechanical properties and anisotropy are reported and justified based on experimental data and theoretical simulation of bi-material parts formed by fully-cured resin and partially-cured resin. Three photopolymers were tested under different working conditions, concluding that post-curing can, in some cases, correct the anisotropy, mainly depending on the nature of photopolymer.

## 1. Introduction

**Figure 1:**
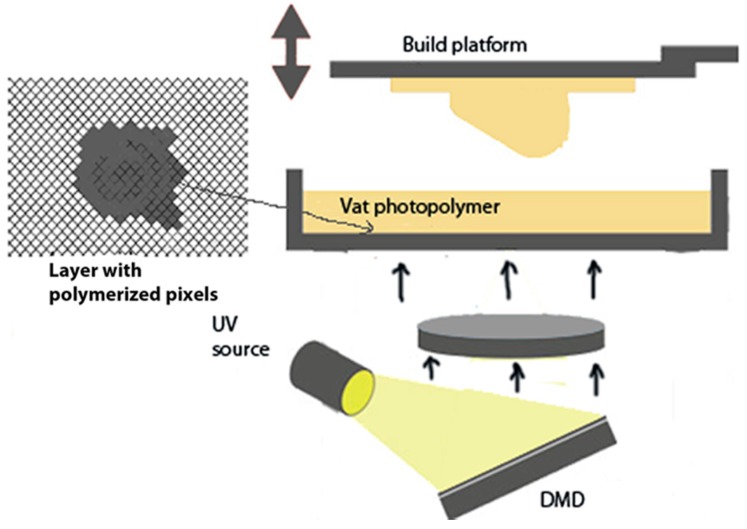
Outline of digital light processing (DLP).

According to ISO/ASTM 52900:2015 [1], Additive manufacturing (AM) is defined as processes of joining materials to produce objects layer upon layer from 3D model data, as opposed to subtractive manufacturing fabrication methodologies. Photopolymers are well-known materials in the field of AM, being Stereolithography (SL) and Polyjet the most common technologies [2], included in the group of vat photopolymerization and material jetting respectively, according with ISO/ASTM 52900:2015. In the last few years, in this particular AM group of vat photopolymerization, Digital Light Processing (DLP) has become an affordable procedure, compared to other expensive AM processes, and suitable for small accurate parts applicable to several sectors requiring micro-parts with small features and complex shapes. DLP is a device based on micro-electro-mechanical technology that uses a digital micro-mirror device (DMD). It was originally developed in 1987 by Dr. Larry Hornbeck of Texas Instruments [3]. The application of this process to AM with photopolymers is based on an array of micro-mirrors suitable for orientation into two positions, moved by micro-actuators. The UV light is reflected by these micro-mirrors to the layer of the liquid photopolymer, placed in a tank. Depending on the orientation of the micro-mirror, each pixel of material is cured or not. The resolution of the cured mask is defined by the number of pixels (Figure 1).

Some applications for DLP parts are the production of microfluidic components (such as valves, pumps, or 3-to-2 multiplexers with an integrated pump) [4], retro-reflective structures (useful for traffic devices, interferometers, and other optical applications) [5], or electromechanical structures (such as capacitors or other parts for electronic devices) [6].

A limitation of photopolymer technologies is the short range of available materials, which should be photo-curable stable resins with optical transparency; furthermore, light penetration should be enough to guarantee good adherence between layers and good part resolution.

It is well known that, in general, AM technologies provide parts with mechanical properties depending on build parameters. In the particular case of laser sintering and stereolithography, the process parameters (laser power, layer thickness, speed hatching, etc.) have shown great influence on performance [7]: In laser sintering, even the amount of recycled powder used has a strong influence on the final dimensions [8] and properties [9] of the part. Otherwise, several studies have focused on the phenomenon of anisotropy as reported by Ahn et al. [10,11] for the technology of material extrusion (commonly known as FDM, Fused Deposition modeling), showing influence of the build direction on mechanical properties of the part. Ajoku et al. [12] reported that polyamide 12 (PA12) under selected laser sintering (SLS) shows anisotropy between vertical and horizontal directions, obtaining the worst mechanical properties when samples were built in the Z direction. Nevertheless, other studies developed by Majewski and Hopkinson [13] do not achieve similar conclusions for sintered PA12, showing almost isotropic behavior. These opposite results are due to the low level of anisotropy of laser sintering compared to other AM processes [14], where under optimal build parameters (recommended by manufacturers of SLS equipment), the material could be almost isotropic. Only when SLS powder is mixed with non-polymeric powder (as glass fiber), does this technology show significant anisotropy [15].

In the field of AM technologies with photopolymers, anisotropy is a typical characteristic, often the anisotropic features obtained are differently from those present when the parts are made with other technologies. Some authors have investigated the influence of build direction on parts made by material jetting technology [16,17,18,19] and stereolithography [20,21,22]. In this sense, Puebla et al. [20] manufactured samples in a Viper SL solid-state laser system using a commercial photopolymer (WaterShedTM), with 30 min post-curing under UV (on a side). Results show better mechanical behavior for parts manufactured on an edge or vertically than built horizontally. This is explained as the influence of SL vectors used to scan the cross-sectional area of the samples. Dulieu-Barton and Fulton [22] found that parts manufactured in an SLA 250 fabricated in vertical orientation provided the highest stiffness, while the ones produced in a horizontal configuration were the weakest ones; they explain this by the over-curing through the layers due to the orientation. Cazón et al. [19] studied the effect of printing orientation and post-processing on the mechanical and surface properties of parts for Polyjet. They found that critical load direction should be placed along the x-axis, and on the xy plane to reduce roughness. Also, Blanco et al. [23] state that UV exposure time is the main parameter affecting the final mechanical properties of the part. They manufactured test samples by using the resin PolyJet RGD240 with Object 30 equipment (Stratasys) and found that elastic modulus is maximal for horizontal parts, which is reduced progressively to a minimum for 60°–75° orientation, while vertical slopes (90°) show an important increase in this parameter. As a conclusion, Blanco states that vertical surfaces with no supports should be more resistant than parts manufactured with supports or in other orientations.

In the end, a general review of all AM technologies demonstrates that most of the parts show anisotropy, from the lowest level (in SLS) to the highest ones (in Laminated Object Manufacturing, LOM) [14], including AM for metals [14].

In order to compare the anisotropy of some of these technologies and materials, Table 1 lists AM technologies/materials with the corresponding ratio between tensile E modulus of samples made in vertical direction ZXY [24] and the ones made in horizontal direction XYZ (rate suitable to measure the level of anisotropy). These values were extracted from the data sheets of manufacturers of AM materials. As observed, some of these manufacturers provide E modulus for ZXY and XYZ direction, while others provide a maximum and minimum value (this explains the different rate calculated depending on the material). In any case, as demonstrated in Table 1, anisotropy is a characteristic declared by manufacturers as well, beyond the work carried out in several studies, as mentioned before. This circumstance is relevant for works carried out by committees for standardization in AM, in ISO, ASTM, and CEN, when developing specific standards for these technologies, which have to take into account anisotropy among other characteristics of AM parts [25].

Not many references about anisotropy in DLP parts have been found, probably because this technology is newer than stereolithography or Polyjet. Since DLP produces pixelated layers, the resolution is a key issue to improve. Riahi [5] studied the optimum parameters to manufacture a cube array, varying layer thickness and building orientation. They found that best results, in terms of roughness, were obtained if the part is oriented at 54.7° around the y direction and 45° around the z direction. Some authors, such as Zoy and Chen [26], developed a calibration method for capturing the non-uniformity of a projection image by a low cost off-the-shelf DLP projector. In this work, the calibration of pixel properties was conducted in two steps, geometric and energy calibrations. In the geometric calibration, magnified pictures are taken and the geometric parameters of a pixel are computed based on image analysis. In the energy calibration, the light intensity was indirectly calculated by using a photosensor. Also, both authors have developed an alternative methodology to reduce the problem of a limited number of micro-mirrors in DMD, therefore conducting to a limited resolution. In this novel method, for each layer, a set of mask images, instead of a single image, are planned based on optimized pixel blending [27]. The planned images are then projected in synchronization with the small movement of the build platform, improving the XY resolution. Kang et al. [28] developed a pixel-based solidification model for projection—based on stereolithography; the intensity profile of a 2D-mask was expressed in terms of a mathematical model, calculating the energy to estimate the solidified profile.

Nevertheless, several works have been carried out to improve mechanical properties of DLP parts. For instance, Peterson et al. [29] varied the light intensity to control cross-link densities, producing functional graded structures. Chiappone et al. [30] investigated hybrid organic-inorganic structures incorporating silica into PEGDA in the presence of photoinitiators under DLP. This innovative combination of unmodified DLP machine and sol-gel technique enabled improvement of mechanical properties. Martin et al. [31] developed discontinuous fiber composite by the technology termed as 3D magnetic printing. This procedure enables distribution and orientation of particles, under a magnetic field, into the photopolymer during DLP process.

In this paper, it is demonstrated that this pixilation in DLP parts is the fundamental cause of anisotropy between vertical and horizontal build directions by experimental testing. The theoretical study of this columnar structure, generated by the pixilation, will provide an estimated value of E modulus of the cured resin placed in the interface of each pixel. Also, how post-curing process can help to reduce this anisotropy is shown.

## 2. Materials and Methods

Three different photopolymer resins have been tested, the first two being colored and commonly applied as pattern models for casting molds (for example dental implants, jewelry, etc.) and the last one is used for models/parts:

- Castable Blend. Monomers: Acrylate/Glycol diacrylate. Photoinitiator: Phosphine oxide based.
- VisiJet^®^ FTX Green. Monomers: Triethylenglycol diacrylate/Tricyclodecane dimethanol diacrylate. Photoinitiator: Phenyl oxide bis (2,4,6-trimethylbenzoyl) phosphine. This resin is supplied into a cartridge designed for Projet 1200.
- Industrial Blend. Acrylate/Glycol diacrylate. Photoinitiator: Phosphine oxide based. This resin was available with and without pigment (insoluble pigment), being this characteristic a key issue for being processed by casting and UV light, as seen later.

In order to characterize the resins under three different processes, three methods have been carried out to manufacture the samples:

- Casting. The resin was poured over a rectangular cavity of a Teflon mold (20 × 30 × 2 mm^3^. To avoid presence of oxygen (which inhibits photopolymerization), a thin film of transparent cellulose acetate was placed on the poured resin. Despite this limitation, Vitale et al. [32] took advantage of this inhibitory effect of oxygen to fabricate multimaterial patterns, using the OIL (oxygen-inhibition lithog-raphy) technology. Afterwards, the resin was cured for 5 min, inside a chamber with UV lamp (Osram Dulux L BL UVA 18W/78 2G11), wavelength 315–400 nm, irradiance 1350 µw/cm^2^ (at 15 cm). Different curing times were tested. Afterwards, different post-curing times to a maximum of 60 min were tested. The UV lamp for post curing was the same as the one for curing.
- Manual layer by layer (MLL). Thin layers (0.15 mm) of resin were spread into a Teflon cavity mold (by brush). Two types of molds with one cavity under the standard ISO 527-1 2012 for tensile samples and the second one with ISO 178:2010 for flexural samples were used. To produce the test bars, 15 layers were necessary. Each layer was cured for 5 min in the chamber with UV lamp, at a wavelength of 315–400 nm and irradiance 1350 µw/cm^2^. Also, a film of transparent acetate was placed on each layer during curing process. The post-curing process took place from 0 to 10 min.
- ProJet™ 1200. DLP equipment (3D Systems corporation, Rock Hill, SC, USA), from 3D Systems, suitable for producing micro-parts with small features. The main characteristics of this equipment are: build platform, 43 × 27 × 150 mm^3^; resolution, 585 ppp (effective); DMD windows designed for transmission region from 320 to 400 nm; layer thickness, 0.03 mm; speed of vertical build, 14 mm/h; average irradiance time per layer, 8.3 s (horizontal sample); software of process and control, Geomagic Print; post-curing chamber UV lamp wavelength, 315–400 nm.

Several replicas of samples were manufactured in Projet 1200 by three strategies of build direction: horizontal (XY), edge (YZ), and vertical (ZX), according with ISO/ASTM52921-13 [24]. In order to facilitate the stability of vertical samples, these were built by placing three parts at 120° joined by removable thin horizontal supports every 10 mm. Also, to observe differences between the locations on the build platform, three locations were selected for horizontal samples: center, back, and front. Since the basic composition and behavior of the Industrial blend and Castable Blend are similar, it was decided to only test the Castable Blend under Projet 1200 and Visijet FTX (the first one with insoluble pigment).

Hardness shore D was measured using a hardness tester, model PCE-DD-D, with resolution 0.5 and accuracy ±2. To measure flatness in the samples, a coordinate-measuring machine (MMC) was used, model Crysta-Plus M443 (Mitutoyo), with 0.0005 mm resolution. Roughness of surface was measured with a roughness instrument Mitutoyo (model SJ-201P).

Tensile and flexural tests were carried out in equipment under a load cell PCE- FB50, with 50 N maximum force, 0.01 N resolution, at 10 mm/min. Since the build platform in Projet 1200 was small, the length of the samples, made in this equipment, could not follow the ISO standard and it had to be cut down to 40 mm (40 × 10 × 2 mm^3^). Nevertheless, the rate between length and thickness is according to ISO standard. In flexural tests, the distance between supports was 30 mm, the radius of supports and punch were 5 mm. For the calculation of ultimate stress and Young modulus, the specific thickness and width of each sample was measured by digital micrometer.

To determine the level of significance of some factors in the different processes carried out, ANOVA analysis and Kruskal-Wallis test were applied. ANOVA analysis provides information about the significance of one factor on the resulting averages of a variable. The Kruskal-Wallis test enables knowledge of the significance of one factor on the medium value of one variable. A P-value below 0.05 means that the corresponding factor is significant on the variation of a variable with a reliability of 95%. Also, a Multiple Range test was implemented. Multiple Range test (MRT) applies a multiple comparison procedure to determine which means are significantly different from which others. In the table of MRT, within each column, the levels containing Xs form a group of means within which there are no statistically significant differences.

In order to demonstrate the difference of stiffness due to the manufacturing orientation (explained and justified in Section 3), flexural properties were simulated by the Finite Element Analysis (FEA). The mesh used was a curvature-based mesh, with a maximum and minimum element size of 1 mm and 0.05 mm, respectively. Parts were defined with the same dimensions as manufactured ones (40 × 10 × 2 mm^3^); double symmetry was applied to simplify the FEA, thus reducing the dimensions to 20 × 5 × 2 mm^3^. On the other hand, two different materials were defined to model the effect of well-cured and non-well-cured resins. According to the measurements in the microscopy, the real dimensions of the well-cured squares were 46 × 46 μm^2^ with 9 μm of separation between squares (shaded areas). However, the definition of this internal structure required the definition of contact boundary conditions between both materials as well as a complex mesh with small elements, which led to problems in the CAD generation and FEA solver. The dimensions of the well-cured squares and shade areas were multiplied by 20 to avoid these problems. Therefore, the internal structure is not the real one, while the proportion between the well-cured and non-well-cured resin is kept. 

## 3. Results

**Figure 2:**
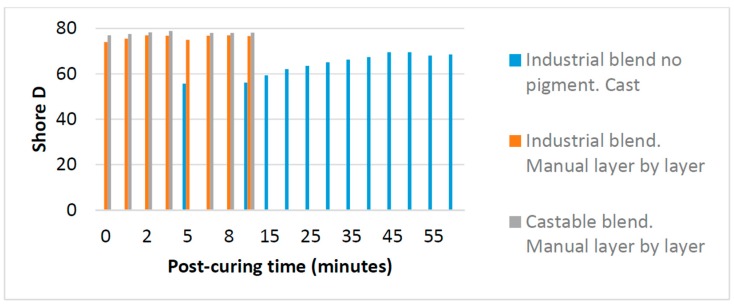
Comparative of shore D and post-curing time, by casting and by manual layer by layer.

Figure 2 shows the influence of post curing time on the hardness Shore D values of parts obtained by MLL. In the case of the resin Industrial Blend, as it is colorless, a comparison between two techniques, casting and MLL, is provided. As expected, casting requires a longer curing time as seen in Figure 2, until about 40 min, when hardness reaches its maximum value. When the applied process is MLL (where each layer is cured separately) post-curing time does not have any effect on increasing the hardness, as observed in Figure 2. Similar conclusions for Industrial Blend and Castable Blend (both with pigments) were observed, with slightly higher value for hardness in Castable Blend.

The MLL methodology is a good reference to compare with automatic DLP 3D printing. Table 2 shows results for tensile test for these resins under casting and MLL, with significant increase of tensile modulus. Note that E modulus of MLL of Castable Blend is even higher than the one under Projet 1200; this is explained by the influence of post-curing and support structures in the Projet-made part, which was post-cured on the build platform (therefore only one side) and with supports. However, without supports, the E modulus in Projet 1200 is improved (Table 2).

Another interesting result (Table 2) is that the flatness error is significantly higher in the case of casting than for LMM. In this one, the post-curing time does not have any influence in this parameter (it is lower than 0.2 mm). It is clear that the casted part, with 2 mm thickness, has important internal stress due to the simultaneous curing process of the entire part thickness, resulting in relevant deformations in the part.

In Project 1200, producing the horizontal samples with supports reduced the mechanical property of samples: the parts manufactured directly on the build platform showed a 40% increase of the tensile modulus, while the flatness error passed from 1.7 to 0.14, after 10 min of post-curing (Table 2). 

**Figure 3:**
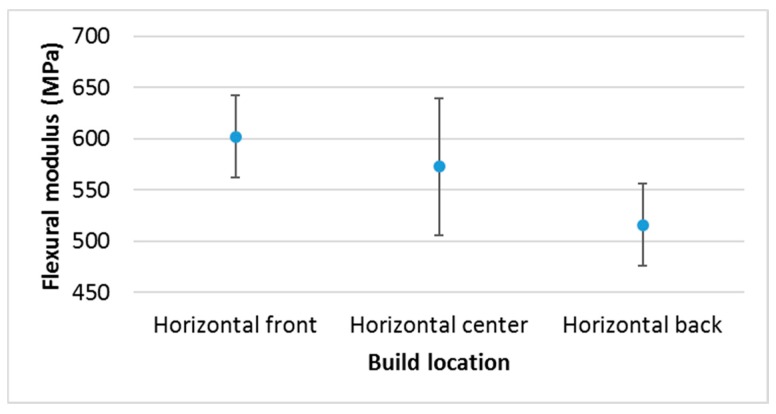
Castable resin in Projet 1200. Different build locations on the platform.

**Figure 4:**
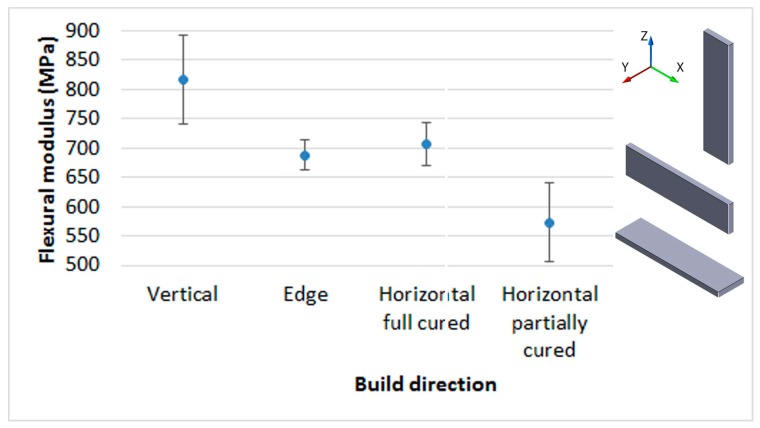
Castable resin in Projet 1200. Different build directions.

The influence of the location of horizontal samples on the build platform (center, back, and front) is shown in Figure 3. The Anova and Kruskal Wallis analyses did not show significant differences among them (*p*-value above 0.05), but the multiple range test (Table 3) demonstrated differences between front and back (within each column, the levels containing Xs or Os form a group of means within which there are no statistically significant differences). Also, Table 3 shows the significant difference between post-curing the horizontal sample on the build platform or doing it on the two opposite faces (seen in Figure 4 as well). It is important to note that the manufacturer of the equipment recommends post-curing the part on the platform, thus reducing the final mechanical properties of the part if the hidden surface is big.

Another crucial issue to study the anisotropy behavior of the photopolymers is the comparison of mechanical properties of samples made at three different build orientations. Figure 4 shows differences for Castable Blend resin, these differences are not significant under Kruskal-Wallis analysis global test (Table 3), although according to Multiple Range Test, differences between horizontally and vertically built samples are significant, both with or without post-curing. Nevertheless, this conclusion is partially similar for the resin Visijet FTX, where the difference between horizontal and vertical is only significant when the resin has not been post-cured. In fact, the values for flexural E modulus are quite similar when post-cured (Table 4).

**Figure 5:**
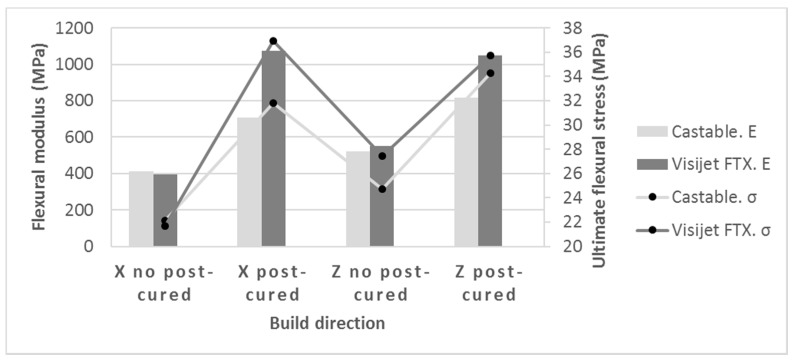
Flexural modulus (E) and ultimate stress (σ). Comparison between resins: Build direction, post-curing, and resin.

As expected, the post-curing process in Projet 1200, increased the mechanical properties of the samples in all the materials and mechanical parameters tested. For example, in Visijet FTX, for horizontal and vertical samples, the ultimate flexural stress was increased over 41% and 77% respectively; for Castable Blend, these increases were 69% and 72% (Figure 5).

## 4. Discussion

The results indicate that for parts made by DLP, vertical samples (Z) provide better mechanical behavior than the horizontal ones (X), before being post-cured (Figure 5). This behavior is different from other AM technologies such as material extrusion (FDM), powder bed fusion (SLS, SLM), LOM, etc., where the vertical sample, due to the build direction of layers, has worse mechanical properties than horizontal ones, both for flexural and tensile tests. Similar results are however shown by some authors: either in stereolithography [20] or in material jetting [14], the photopolymers show better mechanical behavior when parts are made in the vertical direction rather than the horizontal direction. For stereolithography, the explanation for this result is based on the following premises [20]:

- The strength of the adhesion between layers is strong and similar to the adhesion between the material in a layer.
- The type of SL vectors used to scan the cross-sectional area of the sample is one of the main reasons for the difference between the mechanical properties of the samples depending on the build direction. The border vector represents the slower speed and the hatch vector the biggest one. Since the degree of curing depends on the used energy, it is concluded that slower speeds representa higher degree of curing.

The conclusion for material jetting is similar but due to a different reason. Kesy et al. [16] justify this phenomenon because in the polymer jetting technology, UV light hardens each layer. The light emitted by two UV tube bulbs runs parallel to a jet line of the jetting heads. During the jetting process, the sides of created layer parallel to the bulbs absorb more light energy than the layer surface.

**Figure 6:**
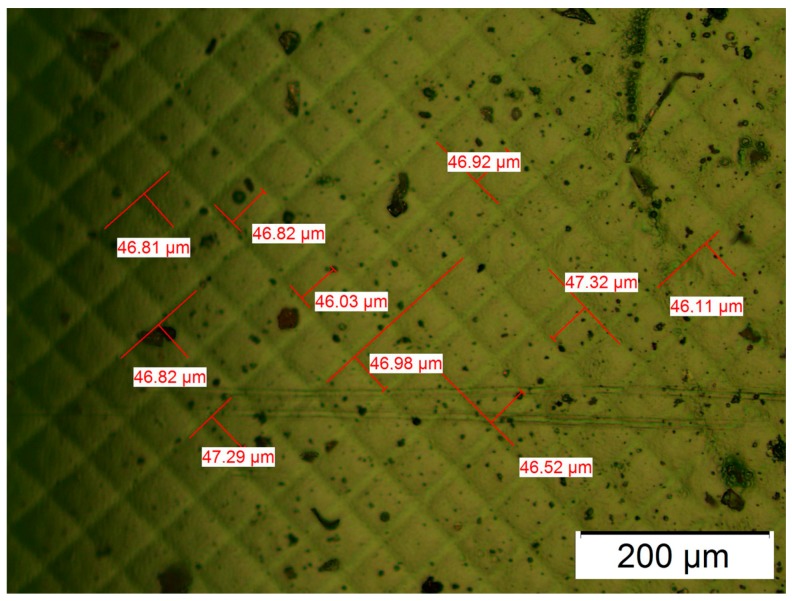
Visijet FTX. Pixilation of DLP part.

**Figure 7:**
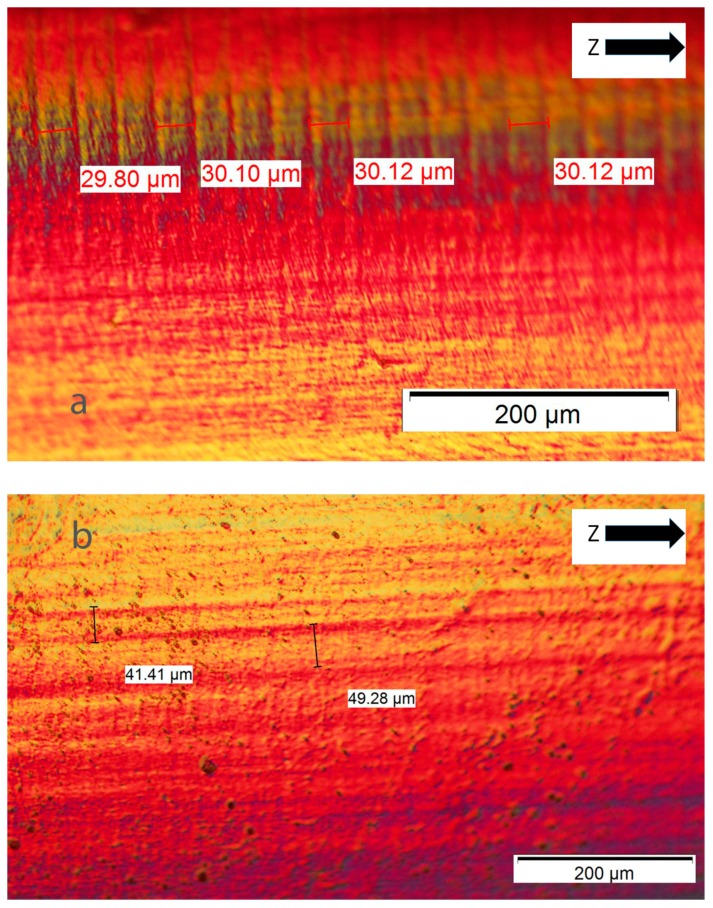
Castable Blend. Fracture section of edge sample. (**a**) Layer thickness; (**b**) Vertical lines due to columns of pixels.

**Figure 8:**
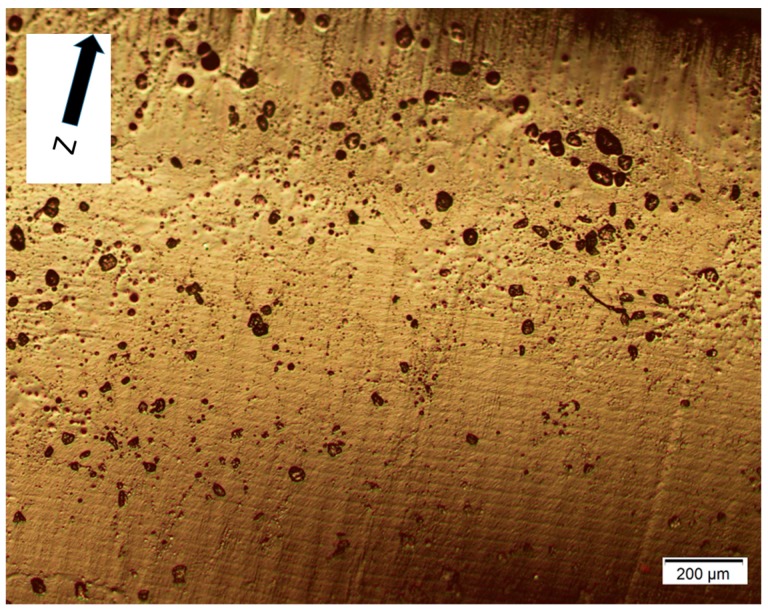
Layers and lines of vertical pixilation in a section of the edge sample. Resin: Castable Blend.

For DLP, the above remarks are not valid except the first premise about strong adhesion between layers. The alternative explanation is based on the resolution of the DLP technology, where the light reflected by each micro-mirror does not completely cure the entire layer, but leaves small areas between each pixel under a poor level of curing. These interstitial areas are due to the separation between micro-mirrors necessary to enable the movement among them. Figure 6 shows a micrograph of the structure of polymerized resin in Projet 1200. The measures of such squares resulted in an average side of 46.8 µm and an average interstitial distance of 9.2 µm. The side of each square (46.8 µm) is according to the theoretical resolution of the equipment Projet 1200 (585 ppp). Figure 7a,b shows two micrographs of the fracture section of Castable resin, where layers about 0.03 mm thick, as declared by the manufacturer, can be observed (Figure 7a) and the parallel lines along the build direction (Z) correspond to the different columns formed by each pixel (Figure 7b). Both types of perpendicular lines are seen in the section of Figure 8, where small holes caused by air bubbles are observed during the polymerization of each layer. An alternative method to demonstrate this characteristic structure of DLP parts was to measure the surface roughness of the lateral face, for example of the vertical sample. The average roughness was 2.3 µm in the X axis and 0.78 µm in the Z direction, according to the previous premise.

**Figure 9:**
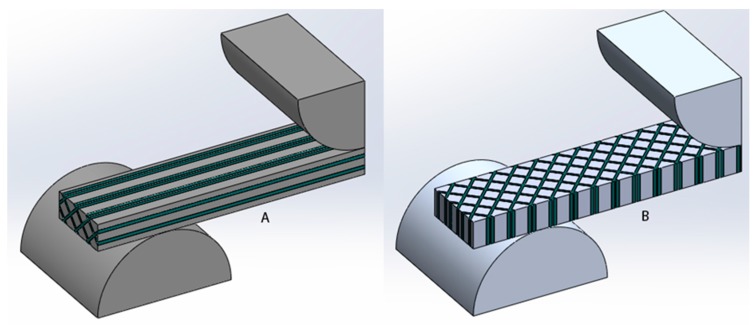
Samples with columns due to pixilation. (**A**) Made under vertical direction; (**B**) Made under horizontal direction.

These premises can explain the different mechanical behavior of vertical and horizontal samples, and therefore the part anisotropy. Figure 9 shows theoretical models of samples for flexural tests manufactured under vertical direction (Z axis) and under horizontal direction (X). The model is based on columns where the material could be considered almost fully cured and interstitial areas where the material has low level of curing. In other words, the part is in fact formed by two materials, as a composite, being the first material with high mechanical properties (fully-cured resin) and the second one with poor mechanical properties (under-cured resin). Under flexural or tensile tests, the sample of Figure 9a is more resistant due to the fiber orientation of stronger material.

**Figure 10:**
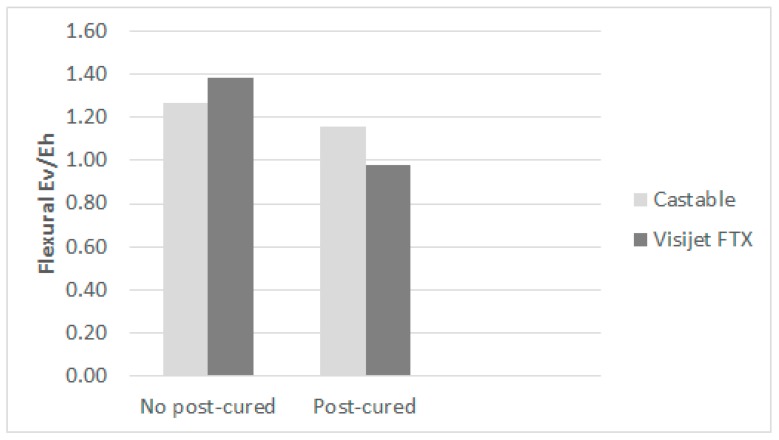
Anisotropy rate. Ev: vertical. Eh: horizontal.

The influence of this structure with columns is relevant depending on the photopolymer and the post-curing process. For example, in Figure 10, it is shown that the rate of anisotropy (rate between E modulus of vertical build direction and the horizontal one). The post-curing process removes the anisotropy in the resin Visijet FTX (from 1.39 to almost 1). This does not happen in the same way with the Castable Blend resin, although this rate changes from 1.27 to 1.16. Taking into account the criterion of Hague et al. [33], for parts without post-curing in both resins, the anisotropy is higher than 5%, so they can be considered as non-isotropic. However, in the post-cured Visijet FTX, made by Projet 1200, the anisotropy is less than 5% and therefore the samples can be considered isotropic. 

The question is why Visijet FTX becomes isotropic when post-cured, while Castable Blend remains non-isotropic. This can be due to the presence of non-soluble pigments in the Castable Blend resin, producing a barrier to UV through interior layers of the part during the post-curing process. This effect is not taking place for Visijet FTX, as the post-curing process is 100% effective.

In terms of final application of the AM part by DLP, the anisotropy could be a problem to deal with, as in some cases post-curing does not solve this problem (as in Castable resin) or post-curing process cannot be performed due to undesirable deformations.

**Figure 11:**
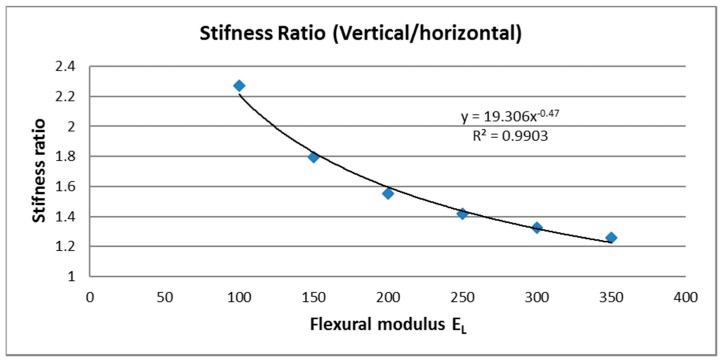
Estimation of flexural E modulus of inter-columnar resin (E_L_). Visijet FTX.

In this context, with no references in AM technologies, the question is: how to design parts for this kind of structure with vertical columns? To do so, the first problem to solve is to estimate the value of the E modulus of fully-cured resin and under-cured resin (inter-columnar) of this composite material. For example, if this methodology is applied to the resin Visijet FTX, the best value for E flexural modulus was obtained when vertical samples were post-cured for 10 min, obtaining a value of 1073 MPa. Figure 11 shows that the E modulus of Visijet, for vertical and horizontal samples, is almost the same when the part has been post-cured. Therefore, the assumption that the fully-cured resin has this maximum value could be an acceptable decision. The only value that needs to be determined to enable simulations of the part by FEA, is the E of inter-columnar area (areas under shadow of UV), when the resin has not been post-cured yet.

To determine this parameter, based on the geometrical model shown in Figure 9, several simulations by FEA were done to calculate the stiffness (force/deformation). Since the E modulus of inter-columnar area (E_L_) was not known a priori, several values of this parameter were supposed to produce a regression curve as shown in Figure 11. The regression equation, shown in Figure 11, allows the prediction of the stiffness of the part depending on the E_L_.

Starting from this equation and considering that the anisotropy rate for Visijet FTX without post-curing is about 1.39 (Figure 10), the resultant flexural E modulus for inter-columnar resin is about 270 MPa. In other words, the theoretical model for mechanical calculation by Finite Element Analysis (FEA), would be based on two flexural modulus. The first E modulus is 1073 MPa (for columns) and the second one 270 MPa for resin between columns. This method would enable prediction of the mechanical behavior of parts without post-curing (Visijet FTX) depending on the build direction on the platform.

## 5. Conclusions

This work has provided knowledge about the anisotropy of photopolymers processed under DLP technology, and has also approached a theoretical model based on considering the final part as a composite formed by two materials with different mechanical properties due to a different grades of polymerization. The model allows the prediction of the mechanical behavior of the part depending on the build direction. The most relevant conclusions are:

- Non-soluble pigments do not enable the casting process due to the difficulty of UV light to go through the full liquid photopolymer.
- The behavior of manual layer-by-layer parts is a good reference to characterize photopolymers, with similar results to the ones obtained with the DLP process.
- In general, the location of the samples on the build platform does not have a significant influence on the mechanical properties.
- The build direction of the part, in particular comparing vertical with horizontal direction, has a significant effect on the mechanical properties, mainly when the post-curing process does not take place.
- The cause of anisotropy in DLP has been justified by a theoretical model of structure with columns of a bi-material composite. The origin of this structure is the pixilation of each layer with shadow areas between pixels, with a poor level of polymerization.
- The post-curing process removes the anisotropy in those resins where the pigment does not obstruct the passing of UV light through the resin.
- In order to simulate the mechanical behavior of DLP parts by FEA under the model of bi-material composite, a regression method is presented to estimate the value of E modulus of the resin placed in the interstitial areas between columns.

## Figures and Tables

**Table 1:** Anisotropy factors in some commercial materials according to technical data sheets. EV: direction ZXY, EH: direction XYZ.

Technology (ISO/ASTM 52900:2015)	Manufacturer	Material	Tensile Modulus Ratio E_V_/E_H_	Tensile Modulus Ratio E_MAX_/E_MIN_
Material extrusion	Stratasys	ABS Plus-P430	0.8	-
Material extrusion	Stratasys	PC	0.9	-
Material extrusion	BQ	PLA	0.9	-
Powder bed fusion of plastic	EOS	PA2200	1	-
Powder bed fusion of plastic	EOS	PA3200 GF	0.8	
Vat photopolymerization	3D System	Accura Amethist	-	1.1
Material jetting	Stratasys	Digital ABS	-	1.2
Powder bed fusion of metal	EOS	Aluminum AISI 10 Mg	0.9	-

**Table 2:** Tensile modulus and flatness error under different post-curing times.

▱ Flatness Error (mm)			Post-Curing Time (Minutes)			
E Tensile Modulus (MPa)			0′	10′	20′	40′
Industrial Blend (no pigment)	Casting	▱ (mm)	0.75		1.23	1.78
		E (MPa)				511.1
Industrial Blend	MLL	▱ (mm)		0.19		0.2
		E (MPa)		609.1		
Castable Blend	MLL	▱ (mm)		0.20		
		E (MPa)		719.0		
Castable Blend with support	Projet1200	▱ (mm)	0.3	1.7		
		E (MPa)		651.2		
Castable Blend no support	Projet 1200	▱ (mm)	0.14	0.14		
		E (MPa)		911.6		

**Table 3:** Statistical study for resin Castable Blend in system DLP Projet 1200.

Resin Castable Blend Statistically Significant Difference between	Anova Analysis *p* Value	Kruskal Wallis Test *p* Value	Multiple Range Test	
Horizontal samples with different location on the build platform	0.055	0.065	Front	X
			Centre	X O
			Back	O
Horizontal samples with partial post-curing and full post-curing	0.020	0.025	Partial	X
			Full	O
Horizontal, edge, and vertical samples, no post-curing	0.049	0.062	Horizontal	X
			Edge	X O
			Vertical	O
Horizontal, edge, and vertical samples, post-cured	0.053	0051	Horizontal	X
			Edge	X
			Vertical	O

**Table 4:** Statistical study for Visijet FTX green in system DLP Projet 1200.

Resin Visijet FTX Green Statistically Significant Difference between	Anova Analysis *p* Value	Kruskal Wallis Test *p* Value	Multiple Range Test	
Horizontal and vertical samples, no post-cured	0.013	0.034	Horizontal	X
			Vertical	O
Horizontal and vertical samples, post-cured	0.732	0.827	Horizontal	X
			Vertical	X
